# Pharmacokinetics of hydrogen after ingesting a hydrogen-rich solution: A study in pigs

**DOI:** 10.1016/j.heliyon.2021.e08359

**Published:** 2021-11-11

**Authors:** Genki Ichihara, Yoshinori Katsumata, Hidenori Moriyama, Hiroki Kitakata, Akeo Hirai, Mizuki Momoi, Seien Ko, Yoshiki Shinya, Kenichiro Kinouchi, Eiji Kobayashi, Motoaki Sano

**Affiliations:** a Department of Cardiology, Keio University School of Medicine, 35 Shinanomachi, Shinjuku-ku, Tokyo, 160-8582, Japan; b Center for Molecular Hydrogen Medicine, Keio University, 2-15-45 Mita, Minato-ku, Tokyo, 108-8345, Japan; c Institute for Integrated Sports Medicine, Keio University School of Medicine, 35 Shinanomachi, Shinjuku-ku, Tokyo, 160-8582, Japan; d Department of Nephrology, Endocrinology and Metabolism, Keio University School of Medicine, 35 Shinanomachi, Shinjuku-ku, Tokyo, 160-8582, Japan; e Department of Organ Fabrication, Keio University School of Medicine, 35 Shinanomachi, Shinjuku-ku, Tokyo, 160-8582, Japan

**Keywords:** Animal experimentation, Translational medical research, Catheterization, Portal venous blood sampling, Cannulation into the jejunal vein

## Abstract

Drinking hydrogen (H_2_)-rich water is a common way to consume H_2_. Although many studies have shown efficacy of drinking H_2_-rich water in neuropsychiatric and endocrine metabolic disorders, their authenticity has been questioned because none examined the associated pharmacokinetics of H_2_. Therefore, we performed the first study to investigate the pharmacokinetics of H_2_ in pigs given an H_2_-rich glucose solution with the aim to extrapolate the findings to humans. We inserted blood collection catheters into the jejunal and portal veins, suprahepatic inferior vena cava, and carotid artery of 4 female pigs aged 8 weeks. Then, within 2 min we infused 500 ml of either H_2_-rich or H_2_-free glucose solution into the jejunum via a percutaneous gastrostomy tube and measured changes in H_2_ concentration in venous and arterial blood over 120 min. After infusion of the H_2_-rich glucose solution, H_2_ concentration in the portal vein peaked at 0.05 mg/L and remained at more than 0.016 mg/L (H_2_ saturation level, 1%) after 1 h; it also increased after infusion of H_2_-free glucose solution but remained below 0.001 mg/L (H_2_ saturation level, 0.06%). We assume that H_2_ was subsequently metabolized in the liver or eliminated via the lungs because it was not detected in the carotid artery. In conclusion, drinking highly concentrated H_2_-rich solution within a short time is a good way to increase H_2_ concentration in portal blood and supply H_2_ to the liver.

## Teaser

Animal experimentation, translational medical research, catheterization, jejunal vein, portal venous blood sampling

## 1 Introduction

Drinking hydrogen (H_2_)-rich water is considered to be a simple way to get H_2_ into the body, and such water is therefore widely consumed for wellness purposes. Many clinical studies found that drinking H_2_-rich water helps maintain health and prevent and treat diseases. For example, drinking H_2_-rich water improved fatigue in healthy individuals [1] and suppressed the rise of lactic acid and reduced muscle fatigue after exercise in elite athletes [2]. H_2_-rich water also improved blood flow-dependent vasodilatory responses in humans [3] and lipid and glucose metabolism in patients with type 2 diabetes or impaired glucose tolerance [4] and lowered cholesterol in people with borderline abnormal lipid metabolism [5]. It improved appetite and taste disorders and suppressed oxidative stress in the blood in radiotherapy patients with liver cancer [6] and was shown to be effective in periodontal disease [7], Parkinson's disease [8], and mild cognitive impairment [9].

H_2_ is an inert gas and does not react without a catalyst, even in the presence of oxygen. It is assumed to have no effects in healthy areas within the body but to intervene in various reactions “where hydroxyl radical damage has occurred” [10]. Therefore, a number of issues have been raised regarding the biological effects of H_2_-rich water, including whether the H_2_ in the water disappears in the stomach; if it does not disappear in the stomach and is absorbed from the intestinal tract, whether it reaches the target organ; and whether it stays in the body long enough to have any effect. The human large intestine actually produces up to 13 L/day of H_2_, about 60%–70% of which is not used by other microorganisms and is excreted via exhalation and intestinal gas [11]. This finding leads to the question whether we can expect any additional effects from drinking H_2_-rich water.

Much basic and clinical research has investigated the effects of H_2_, but no study has provided data on the pharmacokinetics of H_2_ after drinking H_2_-rich water. Therefore, many people are skeptical about the effectiveness of H_2_-rich water. Even if mice were to drink H_2_-rich water ad libitum or H_2_-rich water were to be orally administered to rats' stomachs by a gavage, the findings could not be extrapolated to humans, in particular to when humans drink 300–500 mL of H_2_-rich water within a short time. Furthermore, obtaining pharmacokinetics data in mice and rats from blood samples taken from various parts of the body over time is extremely difficult because of the animals’ small size.

Because of the difficulties of performing pharmacokinetics studies of H_2_ in rodents and the unresolved issues mentioned above, we examined the pharmacokinetics of H_2_ in experimental pigs, which are highly similar to humans in terms of body size and physiology. We aimed to elucidate whether H_2_ from an H_2_-rich solution is absorbed from the small intestine and transported into the bloodstream and whether, after passing through the lungs, it remains in the bloodstream and reaches the arteries.

## 2 Materials and methods

### 2.1 Animals

The present study was designed according to the principles of the ARRIVE (Animal Research: Reporting of In Vivo Experiments) guidelines and was performed in accordance with our institutional guidelines and the Japanese law on the protection and management of animals. Ethics approval was granted by the Research Council and Animal Care and Use Committee of Keio University (approval no: 20005 [1]). The study included 4 female laboratory pigs (ZEN−NOH PREMIUM PIG, Tokyo Laboratory Animals Science Co., Ltd.) aged 8 weeks and weighing 22.5 kg, 22.5 kg, 22.0 kg, and 21.4 kg. The pigs were housed in separate cages under temperature- and light-controlled conditions (12-hour light/dark cycle) and provided with food and water ad libitum. For the 48 h before surgery, they were fed only nutritional milk (Lebens, Wakodo, Japan) to ensure that no food residue was present in the small intestine on the day of the experiment. After induction of anesthesia, the pigs were intubated, and anesthesia was maintained with isoflurane.

### 2.2 Placement of percutaneous gastrostomy tube

**Figure 1:**
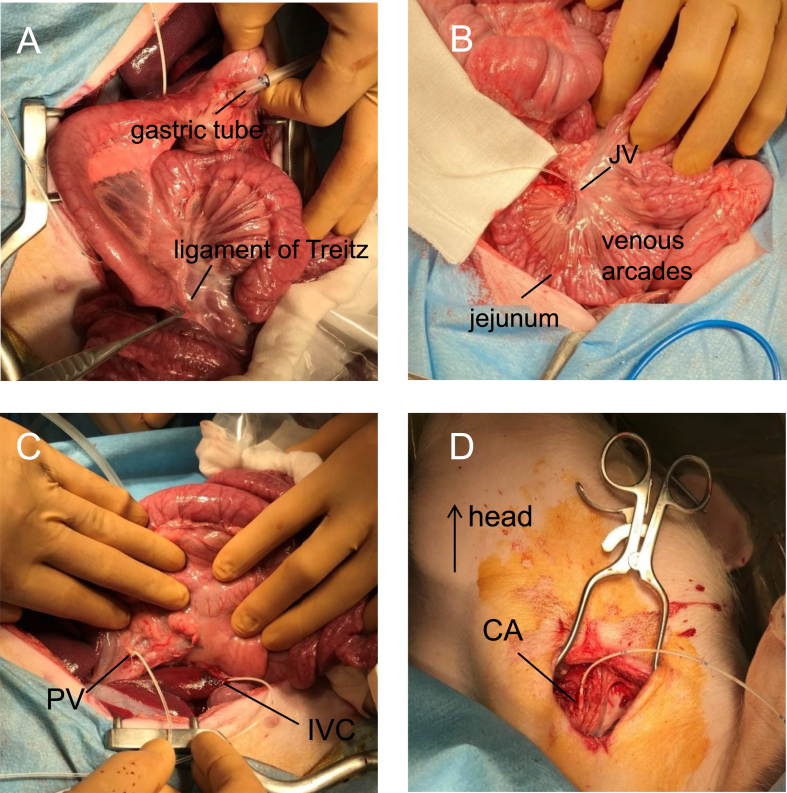
Insertion of blood collection catheter and gastric tube. A. Insertion of gastric tube: The position of the forceps indicates the ligament of Treitz. B. Cannulation of the jejunal vein (JV). The mesenteric serosa of the upper jejunum was dissected into a wedge shape, and the jejunal veins, which form the venous arcades, were dissected out. To avoid congestion, we inserted the catheter into the jejunal vein after checking the arcade of the vein. Notice that the color of the intestine and mesentery is unchanged. C. Cannulation of the portal vein (PV) and inferior vena cava (IVC). D. Cannulation of the internal carotid artery (CA).

To place the percutaneous gastrostomy tube, a 1-cm tobacco-pouch suture was first made with 3-0 Vicryl suture in the anterior wall of gastric antrum. Then, we made a 1-cm incision in the center of the suture, inserted the gastric tube (Covidien Salem Sump, Cardinal Health, Medical Device Certification No. 225AABZBZX00046000), manually guided the tube so that the tip was just past the ligament of Treitz and fixed the tube in place (Figure 1A).

### 2.3 Catheterization of blood vessels

We assumed that H_2_ would be absorbed from the upper jejunal region and flow through the portal vein to the liver. Therefore, before administering the H_2_-rich solution, we inserted blood collection catheters into the jejunal and portal veins and the suprahepatic inferior vena cava. We also inserted a blood collection catheter into the carotid artery to verify whether H_2_ passed through the lungs into the arterial blood. Catheterization was performed as follows:

#### 2.3.1 Jejunal vein

We dissected out the vein that collects blood from the upper jejunal mucosa on the anus side from the tip of the percutaneous gastrostomy tube (jejunal vein) and placed an 18-gauge (18G) central venous (CV) catheter (outer diameter [OD], 1.1 mm) in the direction of the intestine by using our previously developed curved needle guide method [12]. To enable changes in H_2_ concentration to be monitored in real time, we ensured that the tip of the catheter would not cause congestion in the venous arc (Figure 1B). In all pigs, we confirmed that the catheter was in the correct position by assessing the change in glucose concentration in the blood collected from the catheter tip.

#### 2.3.2 Portal vein

We exposed the pancreatic head vein that flows into the portal vein, cut the bifurcation and fed a 16G CV catheter (OD, 1.5 mm) approximately 2.5 cm into the portal vein (Figure 1C).

#### 2.3.3 Inferior vena cava

We made a tobacco-pouch suture of about 5 mm in diameter with 5-0 nylon and partially clamped the vessel with Satinsky vascular forceps. Then, we made an incision in the center of the suture with ophthalmic scissors, inserted a 16G CV catheter (OD, 1.5 mm) and fed the cephalad 18 cm into the inferior vena cava above the liver (Figure 1C).

#### 2.3.4 Carotid artery

First, we made a longitudinal skin incision of approximately 4 cm 1.5 cm lateral to the midline on the left side of the neck. After exposing the left internal carotid artery, we ligated the head side and inserted a 16G CV catheter (OD, 1.5 mm) approximately 5 cm into the aortic arch side and fixed it with 1-0 silk thread (Figure 1B).

### 2.4 Preparation of H_2_-rich solution

**Figure 2:**
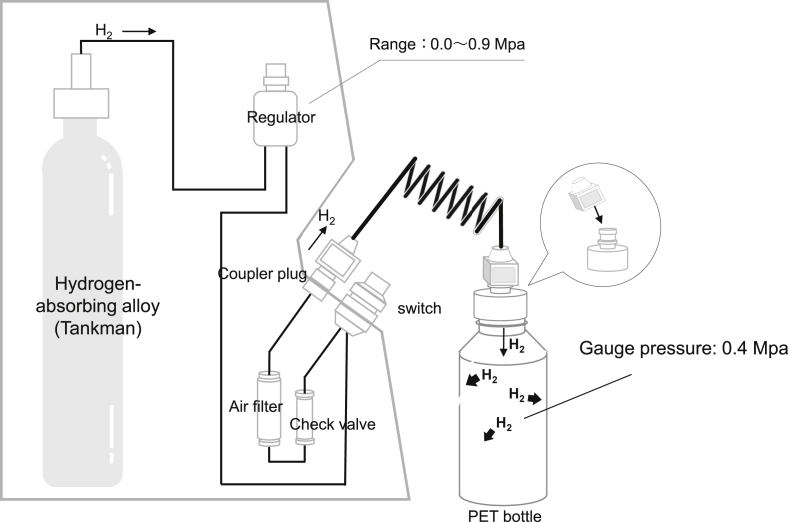
Structure of the hydrogen filling machine. Hydrogen (H_2_) is injected into the polyethylene terephthalate (PET) bottle at the desired pressure by connecting the PET bottle (the cap of which has been replaced with one that integrates a coupler plug with a stop valve) to the coupler socket with a stop valve on the main body of the hydrogen gas filling system. The H_2_ released from the hydrogen storage alloy in the hydrogen gas filling system can be adjusted to the desired pressure by a regulator (pressure control device).

A polyethylene terephthalate (PET) bottle was filled with glucose solution (1.5 g/kg); then, H_2_ was pressurized with a DAYS hydrogen gas filling system (Doctors Man Co., Ltd.) and filled into the bottle to a gauge pressure of 0.4 MPa (Figure 2). The bottle was shaken for 30 s to dissolve the H_2_, and the bottle lid was opened to reduce the pressure to atmospheric pressure. In the equilibrium state, in which the liquid and gas phases coexist in a closed container, the concentration of dissolved H_2_ in the liquid phase depends on the pressure of H_2_ gas in the gas phase; studies have confirmed that 1.6 mg/L of H_2_ can be dissolved in water under an H_2_ gas pressure of 0.1 MPa. The concentration of dissolved H_2_ in the glucose solution is considered to be almost the same as in water. Therefore, when the H_2_ dissolved in the glucose solution, the concentration was expected to be 6.4 mg/L because the H_2_ gas pressure was 0.4 MPa. The reason for agitating the PET bottle was to maximize the contact area between the liquid and the H_2_ gas to dissolve the H_2_ gas efficiently (i.e., to shorten the dissolution time). Stirring may have accelerated the loss of dissolved H_2_ into the air layer, but this was not a problem because the volume of air in the PET bottle was very small compared with the volume of solution. The actual concentration of H_2_ gas in the solution immediately after the PET bottle was stirred for 30 s was 5.42 mg/L (340%). A total of 500 mL of the solution was transferred to a syringe and injected into the small intestine of the pigs through the percutaneous gastrostomy tube over a period of 2 min.

### 2.5 Measuring H_2_ concentration

To measure blood H_2_ concentration, we first inserted a needle into the rubber lid of a 13.5-mL sealed vial, extracted 1 mL of air and injected 1 mL of blood. To prevent outgassing, we immediately applied wax to the rubber lid to seal the injection hole. H_2_ in the blood was released into the air phase in the closed vial. The air in the vial into which blood was initially injected contained almost no H_2_ gas, so most of the H_2_ gas moved from the blood (liquid phase) into the air (gas phase). Therefore, examining the concentration of H_2_ gas in the air inside the vial enabled the H_2_ concentration in the blood to be estimated. Some of the air phase (0.2 mL, 0.4 mL, or 1 mL, depending on the H_2_ concentration) was collected from the vial, and the H_2_ concentration was measured by gas chromatography (TRIlyzer mBA-3000, Taiyo, Co., Ltd.). A calibration curve was obtained by using standard H_2_ gas of 0 (nitrogen gas), 5, 50, and 130 parts per million (ppm). Samples were collected after 2, 5, 10, 20, 30, 40, 50, 60, 90 and 120 min, and each sample was measured twice. At the same time, air was collected from the exact same location into a blood-free vessel, and the H_2_ gas concentration was examined. The value of H_2_ in the air was 0.5 ppm, which exceeds the limit of quantitation. Each measured value of the sample was obtained after subtracting the air value.

## 3 Results

### 3.1 Placement of the catheter in the jejunal vein

In the two pigs injected with H_2_-rich glucose solution and one of the pigs injected with H_2_-free glucose solution, the respective glucose concentrations in the blood sampled from the jejunal vein increased to 221, 251, and 253 mg/dL at 2 min and 317, 397, and 585 mg/dL at 20 min after injection of the solution, indicating that in these pigs the catheter had been correctly placed. In contrast, in the other pig injected with H_2_-free glucose solution the glucose concentration was 71 mg/dL at 2 min and 132 mg/dL at 20 min after injection, indicating that the catheter had not been correctly placed.

### 3.2 Change in blood concentration of H_2_ over time

**Figure 3:**
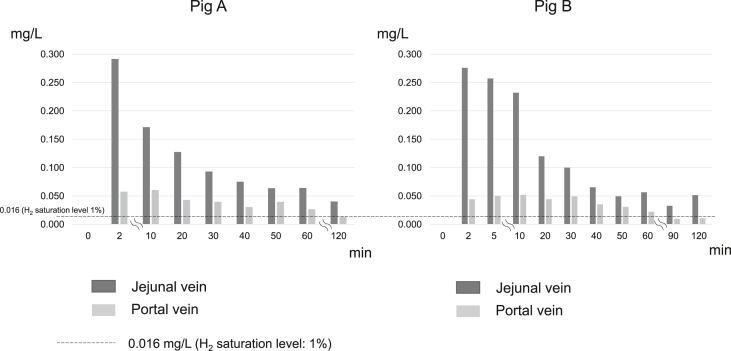
Comparison of the time course of the hydrogen concentration in the jejunal and portal veins after administration to the small intestinal of hydrogen-rich glucose solution. Results from 2 pigs (A, B) treated with hydrogen-rich glucose solution are shown. H_2_, hydrogen.

The data from the two pigs injected with H_2_-rich solution are shown in Figure 3 and Table 1. In these pigs, H_2_ concentrations in the jejunal vein reached 0.292 mg/L (18.3%) and 0.276 mg/L (17.3%) 2 min after injection of the solution into the jejunum. The H_2_ concentration decreased by half after 20 min but was still high after 30 min (0.0930 mg/dL [5.81%] and 0.100 mg/L [6.25%]; both greater than 5%) and after 120 min (0.0402 mg/L [2.51%] and 0.0514 mg/L [3.21%]). In contrast, the peak H_2_ concentration in the portal blood remained at around 0.05 mg/L (∼3%) from 2 to 30 min after the infusion and subsequently gradually decreased; however, even after 60 min it was more than 0.016 mg/L (1%). The H_2_ concentration in the inferior vena cava was about one third lower than that in the portal vein, and no H_2_ was detected in the internal carotid artery.

**Table 1:** **Changes in blood hydrogen concentration in 8-week-old laboratory pigs (N = 2) after administration to the small intestinal of hydrogen-rich glucose solution.** Studies have confirmed that under a hydrogen (H_2_) gas pressure of 0.1 MPa, 1.6 mg/L of H_2_ can be dissolved in water. The H_2_ saturation in blood is shown in % when 1.6 mg/L is considered to be 100% saturation. Each significant digit corresponds to the number of digits of the measurement. When a measured reading was indistinguishable from the background (air), it was shown as "not detected" (n.d.) instead of zero.

Location of measurement		Concentration after administration of H_2_-rich glucose solution								
		Before	2 min	10 min	20 min	30 min	40 min	50 min	60 min	120 min
jejunal vein	mg/L	n.d.	0.292	0.171	0.127	0.0930	0.0750	0.0636	0.0640	0.0402
	%	n.d.	18.3	10.7	7.96	5.81	4.69	3.97	4.00	2.51
portal vein	mg/L	0.00047	0.0574	0.0603	0.0429	0.0395	0.0303	0.0395	0.0265	0.0132
	%	0.029	3.59	3.77	2.68	2.47	1.89	2.47	1.66	0.825
inferior vena cava	mg/L	n.d.	0.0125	0.0157	0.0214	0.0103	0.00935	0.00853	0.00853	0.0048
	%	n.d.	0.782	0.979	1.34	0.643	0.584	0.533	0.533	0.30
carotid artery	mg/L	n.d.	n.d.	n.d.	n.d.	n.d.	n.d.	n.d.	n.d.	n.d.
	%	n.d.	n.d.	n.d.	n.d.	n.d.	n.d.	n.d.	n.d.	n.d.

Location of measurement		Concentration after administration of H_2_-rich glucose solution, mg/L (%)										
		Before	2 min	5 min	10 min	20 min	30 min	40 min	50 min	60 min	90 min	120 min
jejunal vein	mg/L	0.0002	0.276	0.257	0.232	0.120	0.100	0.0652	0.0492	0.0563	0.0326	0.0514
	%	0.1	17.3	16.1	14.5	7.50	6.25	4.08	3.08	3.52	2.04	3.21
portal vein	mg/L	0.00088	0.0441	0.0501	0.0519	0.0442	0.0491	0.0350	0.0305	0.0219	0.0093	0.011
	%	0.055	2.76	3.13	3.24	2.76	3.07	2.19	1.91	1.37	0.581	0.69
inferior vena cava	mg/L	0.0003	0.011	0.0189	0.0198	0.0147	0.0129	0.0127	0.0078	0.0088	0.0030	0.0029
	%	0.02	0.69	1.18	1.24	0.920	0.803	0.796	0.49	0.55	0.19	0.18
carotid artery	mg/L	n.d.	0.0001	0.0001	0.0002	0.0002	0.0001	0.0001	0.0001	0.0001	0.0001	0.0001
	%	n.d.	0.007	0.007	0.015	0.011	0.007	0.007	0.007	0.007	0.004	0.007

**Figure 4:**
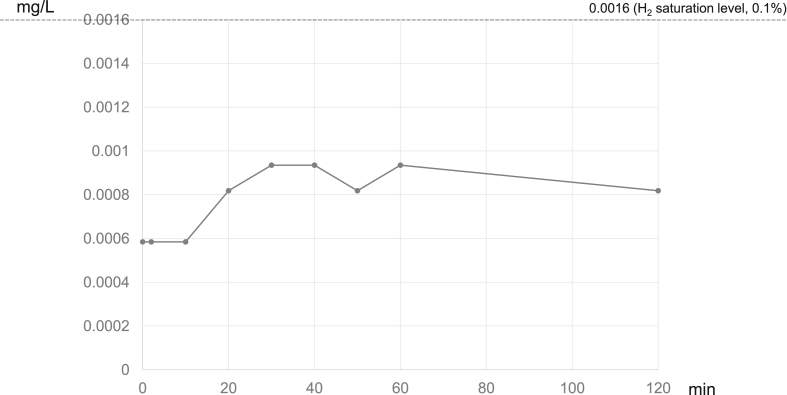
Hydrogen concentration in the portal vein after administration of hydrogen-free glucose solution. H_2_, hydrogen.

Figure 4 shows the change in H_2_ concentration in the portal vein of the pig administered H_2_-free solution in which the catheter was confirmed to be in the correct position. The H_2_ concentration in the portal blood was 0.0005 mg/L (∼0.03%) at baseline; it increased 20 min after administration of the solution, but only to 0.0009 mg/L (∼0.06%).

## 4 Discussion

Although more than 1,600 studies have investigated the biological effects of H_2_, none has assessed the pharmacokinetics of H_2_ in animals with the aim to extrapolate findings to humans. Therefore, we investigated the pharmacokinetics of H_2_ when H_2_-rich glucose solution was injected directly into the small intestine of pigs over a 2-minute period. We found that the H_2_ concentration in the portal vein was less than 0.001 mg/L at baseline, even though gut bacteria could be assumed to be producing large amounts of H_2_, and that infusion into the jejunum of H_2_-rich solution provided sufficient H_2_ to markedly increase H_2_ concentration in the portal vein. Furthermore, we showed that H_2_ concentration in the portal vein was maintained for about 1 h, which may have been due to continued absorption of H_2_-rich solution by the blood as it passed through the jejunum. The concentration in the portal vein was at a level that inhibited ischemia-reperfusion injury in humans [13]. No increase in H_2_ concentration was seen in arterial blood (carotid artery).

In pigs, the large intestine is significantly thicker than the small intestine and contains a large number of bacteria, which digest food that is not digested in the small intestine, especially fiber. While placing the feeding tube and catheters, we confirmed that the large intestine was packed with stool in all 4 pigs. Nevertheless, portal blood contained less than 0.001 mg/L of H_2_, even after administration of H2-rich glucose solution to the jejunum. Previously, we devised a methodology for single inhalation of gas and demonstrated for the first time that inhaled H_2_ is absorbed from the lungs and distributed throughout the body via the blood [14]. In that study, animals were fed a normal diet until 12 h before the experiment, and H_2_ in the portal vein was also below the detection limit [14]. Taken together, this evidence suggests that H_2_ derived from intestinal bacteria is absorbed to a small extent but does not increase the H_2_ concentration in portal blood to a biologically meaningful level. In other words, only by drinking sufficient H_2_-rich solution over a short time can the H_2_ concentration in the blood be increased, albeit locally. In this study, we showed that the H_2_ concentration increases 50-fold (up to 0.05 mg/L) after consuming a solution with highly concentrated H_2_.

We found no change in the H_2_ concentration in the carotid artery. However, our earlier study found a very high concentration of H_2_ in the carotid artery after a single inhalation of the gas, confirming that inhalation is the best way to supply H_2_ to the entire body through the arterial blood [14]. Another study of ours used a different experimental system in which 100% H_2_ was continuously inhaled through a nasal cannula; here, the H_2_ concentration in the arterial blood matched the predicted value of the fraction of inspired H_2_, i.e., the concentration of H_2_ in the gas mixture [15]. This experiment proved that the H_2_ concentration in arterial blood is proportional to the partial pressure of H_2_ in the inhaled gas mixture.

Translating our present results to humans, we suggest that drinking H_2_-rich water is an effective way to supply H_2_ directly to the liver. Nonalcoholic fatty liver disease, which can develop into more severe diseases such as steatohepatitis and cirrhosis, is associated with obesity, diabetes, dyslipidemia, and hypertension, conditions that are considered to be phenotypes of the metabolic syndrome [16]. The development of these pathological conditions involves metabolic abnormalities, activation of the immune system, and increased oxidative stress in the liver [17], and previous research indicates that H_2_ derived from H_2_-rich water may work directly on the liver to produce various beneficial clinical effects [18]. The above-mentioned finding that drinking H_2_-rich water suppresses the rise of lactic acid and decreases muscle fatigue after exercise may be based on the activation of the Cori and glucose-alanine cycles between skeletal muscle and the liver [2]. Drinking H_2_-rich water was also reported to cause gastric secretion of the neuroprotective substance ghrelin [19].

As for the diffusion of H_2_ into the blood, equilibrium can be assumed to be established between the lumen and vascular sides across the epithelium [20]. This process is affected by the thickness of the septum, pressure and surface area in the lumen, and flow velocity of the vessel. The lungs are specialized for gas exchange in that the thin type I alveolar epithelial cells (squamous epithelial cells), which have a thickness of 0.1–0.2 μm, are so close to the capillary wall (endothelial cells) that the two cannot be distinguished under a light microscope. In our above-mentioned experiment with inhaled H_2_, the H_2_ saturation of the arterial blood instantly increased to a level comparable to the H_2_ concentration in the alveoli [14, 15]. However, the intestinal lumen is covered by a single layer of columnar epithelium, so the diffusion distance from the lumen to the capillaries is much greater than that from the pulmonary alveoli to the capillaries. In fact, neither pigs nor humans can perform intestinal respiration. Some of the H_2_ produced by gut bacteria is absorbed into the bloodstream; however, we suggest that the H_2_ concentration in portal blood remains at an extremely low level because of its rapid excretion through exhalation.

This study has some limitations. It proved experimentally that the H_2_ concentration in the portal vein can be increased by pouring a highly concentrated H_2_-rich solution into the intestinal tract within a short period. When humans drink H_2_-rich water, the water can be assumed to stay in the stomach for a certain time [21]. H_2_ is efficiently absorbed via the stomach wall, where the internal pressure is higher than in the small intestinal tract because the pylorus and cardia of the stomach are closed. However, the surface area of the stomach is much smaller than that of the small intestine, where millions of tiny finger-like structures called villi project inwards from the lining. Furthermore, we need to consider the possibility that H_2_ absorption is enhanced in the small intestine along with the absorption of water. Intestinal bacteria are known to generate H_2_ [20, 21, 22, 23, 24], in particular the phyla Firmicutes and Bacteroidetes, the 2 major phyla in the human colon [11]. The gut microbiota is particularly affected by the amount of fiber and fat consumed and changes dramatically even within 24 h. Therefore, in this study the intestinal microbiota and metabolite composition of the pigs’ intestinal tract after ingesting nutritional milk for 48 h may have been significantly different from that with a normal diet. A large amount of stool was still present in the colon, so the colon lumen could be expected to have a significant concentration of H_2_, although no biologically meaningful concentration of H_2_ was detected in the portal blood (less than 0.001 mg/L), even after glucose loading.

To our knowledge, this is the first paper to examine the pharmacokinetics of H_2_ in pigs through the advanced experimental technique of catheterization in the intestinal region. We attempted to extrapolate the findings to humans and believe that to increase the H_2_ concentration in the portal circulation to a substantial level an adequate amount of water with a high concentration of H_2_ must be drunk within a short period. Nevertheless, H_2_ does not appear to reach the systemic circulation. This topic warrants further study in humans because of the hypothesized beneficial effects of H_2_ in the abdominal organs, which will receive a sufficient concentration of H_2_ gas from the intestinal tract through the portal blood flow to the liver.

## Declarations

### Author contribution statement

Genki Ichihara; Yoshinori Katsumata; Hidenori Moriyama; Hiroki Kitakata; Akeo Hirai; Mizuki Momoi; Seien Ko; Yoshiki Shinya; Kenichiro Kinouchi: Performed the experiment.

Eiji Kobayashi: conceived and designed the experiments; performed the experiment; contributed reagents, materials, analysis tools or data.

Motoaki Sano: Conceived and designed the experiments; Performed the experiments; Analyzed and interpreted the data; Contributed reagents, materials, analysis tools or data; Wrote the paper.

### Funding statement

This work was supported by grants from Doctors Man Co., Ltd. (M.S.). Sou Hashimoto (Doctors Man Co., Ltd.) provided us with the H2 filling device. The funders had no role in study design, data collection and analysis, decision to publish, or preparation of the manuscript.

### Data availability statement

Data included in article/supplementary material/referenced in article.

### Declaration of interests statement

Motoaki Sano, and Eiji Kobayashi received advisory fees from Doctors Man Co., Ltd. Furthermore, Motoaki Sano, and Eiji Kobayashi are the registered inventors of the patent jointly filed by Keio University and Doctors Man, “Methods for generating organ preservation solution containing hydrogen and organ preservation solution containing hydrogen” (Application number PCT/JP2019/045790).

### Additional information

No additional information is available for this paper.
